# Association between GNRHR, LHR and IGF1 polymorphisms and timing of puberty in male Angus cattle

**DOI:** 10.1186/1471-2156-13-26

**Published:** 2012-04-05

**Authors:** Juan P Lirón, Alberto J Prando, María E Fernández, María V Ripoli, Andrés Rogberg-Muñoz, Daniel E Goszczynski, Diego M Posik, Pilar Peral-García, Andrés Baldo, Guillermo Giovambattista

**Affiliations:** 1 Instituto de Genética Veterinaria (IGEVET), CCT La Plata - CONICET - Facultad de Ciencias Veterinarias, Universidad Nacional de La Plata, La Plata, Argentina; 2 Departamento de Producción Animal, Facultad de Ciencias Veterinarias, Universidad Nacional de La Plata, La Plata, Argentina; 3 Comisión de Investigaciones Científicas de la Provincia de Buenos Aires, Buenos Aires, Argentina

**Keywords:** Gonadotropin releasing hormone receptor, Luteinizing hormone receptor, Insulin-like growth factor 1, Bovine, Polymorphism, Male puberty

## Abstract

**Background:**

In bovines, there are significant differences within and among beef breeds in the time when bulls reach puberty. Although the timing of puberty is likely to be a multigenic trait, previous studies indicate that there may also be single genes that exert major effects on the timing of puberty within the general population. Despite its economic importance, there are not many SNPs or genetic markers associated with the age of puberty in male cattle. In the present work, we selected three candidate genes, *GNRHR*, *LHR *and *IGF1*, and associated their polymorphisms with the age of puberty in Angus male cattle.

**Results:**

After weaning, 276 Angus males were measured every month for weight (W), scrotal circumference (SC), sperm concentration (C) and percentage of motility (M). A total of 4 SNPs, two within *GNRHR*, one in *LHR *and one in *IGF1 *were genotyped using the pyrosequencing technique. *IGF1-SnaBI SNP *was significant associated (P < 0.01) with age at SC 28 cm, but it were not associated with age at M 10% and C 50 million. Genotype *CC *exhibited an average age at SC 28 cm of 7 and 11 days higher than *CT *(p = 0.037) and *TT *(p = 0.012), respectively. This SNP explained 1.5% of the genetic variance of age of puberty at SC28. *LHR-I499L*, *GNRHR-SNP5 *and *GNRHR-SNP6 *were not associated with any of the measurements. However, *GNRHR *haplotypes showed a suggestive association with age at SC 28 cm.

**Conclusions:**

The findings presented here could support the hypothesis that *IGF1 *is a regulator of the arrival to puberty in male calves and is involved in the events that precede and initiate puberty in bull calves. Given that most studies in cattle, as well as in other mammals, were done in female, the present results are the first evidence of markers associated with age at puberty in male cattle.

## Background

Among cattle breeds, significant differences have been reported in the age when bulls reach puberty [1-5]. Although the timing of puberty is likely to be a multigenic trait, data from human and mouse indicate that there could be single genes with major effects involved. Despite its economic importance, there are few genetic markers associated with this trait. Recently, gonadotropin-releasing hormone receptor (*GNRHR*) polymorphisms have been associated with the days to first service after calving in dairy cattle [6], and with age of first *corpus luteum *in Brahman and Tropical Composite female [7]. Recently, Fortes et al. [8,9], using a system biology approach in Brahman and Tropical Composite dams, identified candidate genes and pathways (*e.g. *axon guidance, cell adhesion, ErbB signaling, and glutamate activity) that are known to affect pulsatile release of *GNRH*. However, these still remain to be investigated in bovine males. This reinforces the importance of identifying genes that regulate puberty and polymorphisms that explain these differences in bulls. To date, it is known that some male traits, such as scrotal circumference at 12 months and IGF1 serum concentration at 6 months, may be correlated with early female reproductive performance, measured as age at first *corpus luteum*. These results suggest that selection toward early puberty males would indirectly reduce the timing of puberty in their female relatives http://www.bifconference.com/bif2011/documents/GenPredJohnston.pdf and http://www.beefcrc.com.au/, accession date: 02/09/12). This correlation would indicate that there are common genes involve in the development of puberty in both sexes.

GNRH and its receptor play a major role in coordinating sexual differentiation and reproduction in mammals, mediating the secretion of gonadotropic luteinizing hormone (LH) and follicle stimulating hormone (FSH). These hormones regulate gonadal function, including steroid hormone synthesis and gametogenesis. Genetic variations within *GNRHR *contribute to the regulation of pubertal timing in human and mouse [10]. In mammals, insulin like growth factor 1 (IGF1) has been identified as a signal controlling reproductive function, with possible links to somatic growth, particularly during puberty [11]. This factor has direct actions on *GNRH *neurons, and influence testicular growth, determining the development of seminiferous tubules and Leydig cells [12]. Furthermore, positive correlations were also found between serum concentrations of IGF1 and serum LH and testicular LH receptor (LHR) concentrations in bull calves [12]. Consequently, *IGF1 *could be considered a strong candidate gene for the regulation of testicular growth in the bull calf during pre-pubertal period. In the present work, we selected three candidate genes *GNRHR*, *LHR *and *IGF1*, to associate their polymorphisms with the age of puberty in male cattle; and we reported the first evidence of a marker associated with age at puberty in male cattle.

## Results and discussion

All alleles were present within both herds, with minimum allele frequency and expected heterozygosity higher than 0.10 and 0.179, respectively. A total of fifteen (ten within population and five for the entire sample) Hardy-Weinberg equilibrium tests were performed. With the exception of *GNRHR *haplotypes in Herd 1 and the combined population, none of these tests exhibited significant deviations from theoretical proportions (P > 0.05) (Additional file 1: Table S1). These results evidenced that all the studied SNPs were suitable for association.

In bovine, several QTLs related to reproductive traits have been detected using microsatellites, some of which were associated with age at puberty http://www.animalgenome.org/cgi-bin/QTLdb/BT/search. Most reported studies have used simpler indirect measurements (*e.g*. scrotal circumference, calculated age at puberty, age at first insemination) [5,6,13,14]. Recently, SNPs have been associated with timing of puberty in females [6-9,13], but to date, they still remain unknown in males.

In the present work, we report the first evidence of markers associated with age at puberty in male cattle. Significant associations were detected between *IGF1-SnaBI *SNP and age at SC 28 cm (p < 0.05), but it were not associated with age at M 10% and C 50 million. Genotype *CC *exhibited an average age at SC 28 cm of 7 and 11 days higher than *CT *(p = 0.037) and *TT *(p = 0.012), respectively. This SNP explained 1.5% of the genetic variance of age of puberty at SC28. *LHR-I499L*, *GNRHR-SNP5 *and *GNRHR-SNP6 *were not associated with any of the measurements. However, *GNRHR *haplotypes showed a suggestive association with age at SC 28 cm (Table 1). A resume of average phenotypic data was detailed in Additional file: Table S2.

**Table 1:** Average estimated puberty age and standard error (SE) in days, for the genotypes of the SNPs *LHR-I499L *and *IGF1-SnaBI*, and the haplotypes of *GNRHR*; and the P-val for the genotype effect in the model are presented for the two criterions for age of puberty are showed: i) estimated at Scrotal Circumference = 28 cm, ii) estimated at Sperm Motility = 10% and Sperm Concentration = 50 million

Marker	Genotypes	N	Age at SC28		Age at 5 10^7^/10% mot	
			**Genotype effect P-val**	**Age ± SE**	**Genotype effect P-val**	**Age ± SE**

LHR-I499L	AA	17	0.456	292.27 ± 6.84	0.301	288.73 ± 11.04
	AC	94		284.87 ± 4.21		287.87 ± 6.89
					
	CC	165		287.28 ± 3.71		279.96 ± 6.12

IGF1-SnaBI	CC	84	0.027	294.27 ± 4.58**A**	0.354	287.55 ± 7.59
	CT	142		287.17 ± 4.28**B**		281.02 ± 6.95
					
	TT	50		282.98 ± 4.94**B**		287.98 ± 8.06

GNRHR haplotypes	GC/GC	22	0.271	300.15 ± 5.90**a**	0.124	291.82 ± 9.63
					
	GC/GT	43		296.75 ± 4.32**a**		288.27 ± 7.09
					
	GC/AC	12		293.00 ± 7.65		277.96 ± 12.69
					
	GC/AT	78		289.79 ± 3.60		288.50 ± 6.11
					
	GT/GT	15		290.43 ± 6.79		262.15 ± 11.09
					
	GT/AT	53		289.93 ± 3.68		291.98 ± 6.30
					
	AC/AC	2		271.02 ± 23.69		322.16 ± 36.18
					
	AC/AT	5		270.23 ± 10.71**b**		256.41 ± 16.80
					
	AT/AT	47		291.97 ± 4.04		290.42 ± 6.99

Different Bold Capital letters denote significant association (p < 0.05) between means. Different bold lower letters denote suggestive association (p < 0.1) between means. Bonferroni's means adjustment was performed for multiple comparisons

Even though *GNRH*, *LH *and its receptors play a major role in coordinating sexual differentiation and reproduction in mammals, and there are strong evidences that genetic variation within these genes contributes to the regulation of pubertal timing in human and mouse populations [15], the present study showed no effect of *GNRHR *and *LHR *SNPs on analyzed puberty traits. Our results are in agreement with a previous work [13] that also did not find correlations between early puberty in Nelore females and mutations in the *LHR *and *GNRHR *genes. Nevertheless, other polymorphisms in linkage disequilibrium within these genes could be associated with the analyzed traits. Further studies are necessary to evaluate the influence of *GNRHR *and *LHR *variations in the events that precede and initiate puberty in bull calves, as well as testis growth and gametogenesis.

Physiological and gene expression data showed that *IGF1 *is involved in the events that precede and initiate puberty in bull calves. Recent evidence showed that IGF1 can regulate the hypothalamic-pituitary-gonadal axis via direct actions at the pituitary and gonadal levels of this axis [11]. In this sense, pre-pubertal IGF-1 serum concentrations have been genetically correlated with adult scrotal circumference and sperm motility, in bulls; and with the age at first calf and calving rate, in females [16]. In mammals, IGF1 has been identified as a regulator of testicular growth, influencing the development of the seminiferous tubules and Leydig cells [12]. Positive correlations were also found between serum concentrations of IGF1 and LH and testicular LHR concentrations in bull calves. Moreover, the *IGF1-SnaBI *has been demonstrated to influence *IGF1 *gene expression and IGF1 blood level in cattle [17]. In addition, IGF1 up-regulates *LHR *and testosterone secretion in Leydig cells, and it has been shown to have an additive effect of LH and IGF1 on testicular cells number. Furthermore, IGF1 influences GNRH neurons during puberty through its receptor (IGF1R). For these reasons, *IGF1 *has been considered a strong candidate gene for the regulation of testicular growth in the bull calf during pre-pubertal period [11].

Regarding this, IGF1-SnaBI SNP was reported to affect animal weight at different ages in cattle [18-21]. However, in bovine and human, puberty has been associated more with stature or BMI than with weight [22,23]. Nevertheless, the effect of weight (estimated W at 300 days) was included as covariate in the used association statistical model. Considering the correlation between these traits, a permissive effect of IGF1on reproductive development, rather than a direct effect as discussed above, could explain the observed association between this SNP and estimated age at SC28 [24]. Regardless of the involved effect (direct or permissive), this findings support the hypothesis that IGF1-SnaBI SNP could be a useful predictor of early age of puberty.

## Conclusions

The findings presented here could support the hypothesis that *IGF1 *is a regulator of the arrival to puberty in male calves and is involved in the events that precede and initiate puberty in bull calves. Given that most studies in cattle, as well as in other mammals, were done in female, the present results are the first evidence of markers associated with age at puberty in male cattle.

## Methods

### Sample collection

The study was performed on 276 Angus males belonging to two herds located in Buenos Aires Province (Argentine). Calves, sired by 24 bulls, were born in 2008 and 2009 winters, between July and September. After weaning and during the whole experiment, animals were pasture fed. Every month, animals were weighed (W) and scrotal circumference measured (SC). When first bull calves reached 26 cm of SC, sperm concentration (C) and percentage of motility (M) were added to the monthly measurements for the next three months. These monthly SC, C and M measurements were similar to those made by Lunstra and Cundiff [4], and are adequate to get the meaningful changes during the peri-pubertal development.

The present research was carried out following the internationally recognized guidelines, and the experimental design was approved by the "Comité Institucional para el Cuidado y Uso de los Animales de Laboratorio" (Faculty of Veterinary Sciences, National University of La Plata, Argentina; Res N° 129/09).

#### Scrotal circumference and sperm quality measurements

Scrotal circumference (SC) was measured with a flexible measuring tape at the greatest horizontal distance around the scrotum after manually forcing the testicles into the base of the scrotum, as described by Kealey *et al *[25]. Semen samples were collected by electroejaculation. Sperm concentration (C) were measured using a photometer SPERMACUE 12300/0500 (Minitube, Tiefenbach, Germany), while percentage of progressive motility (M) were calculated by duplicate estimates on semen diluted in phosphate buffered saline (pH 7.4), using a microscope (×400 magnification) equipped with a stage warmer (37°C) [26].

#### DNA extraction and pyrosequencing genotyping

DNA was extracted from blood samples using Wizard Genomic kit following manufacturer instructions (Promega, Madison, WI, USA). Herein, we selected four polymorphisms located into three candidate genes: two SNPs in *GNRHR *and one in *IGF1 *that were previously reported by Lirón *et al. *[27] and Ge et al. [18], respectively. The *GNRHR-SNP5 *and *GNRHR-SNP6 *were chosen because a previous study showed that these SNPs were sufficient to tag the four more frequent *GNRHR *haplotypes present in the studied Angus populations [27]. The *IGF1-SnaBI *SNP, located in the promoter region, was selected because TESS (Transcription Element Search System; http://www.cbil.upenn.edu/cgi-bin/tess/tess?RQ=WELCOME) analysis showed that it co-localizes with the NF1 transcription factor binding site [17]. *LHR-I499L *corresponds to a novel SNP detected in the studied Angus populations by re-sequencing analysis of *LHR *exon 11. This exon was analyzed because it codifies for a transmembrane region of the protein, a critical domain for the receptor function. Despite this SNP has not been associated yet with the age of puberty in male cattle, it has been reported that mutations on this region of LHR can cause sexual precocity in humans, which justifies the search of polymorphisms in this exon [28].

Four pyrosequencing assays were developed to genotype *GNRHR-SNP5 *[19], *GNRHR-SNP6*, *LHR-I499L*, and *IGF1-SnaBI *SNPs. First, PCR was carried out for each gene in a total volume of 50 μl, containing 20 mM Tris-HCl (pH = 8.4), 50 mM KCl, 2.5 mM MgCl2, 100 mM of each dNTP, 0.75 U Taq polymerase (Metabion, Martinsried, Germany), 0.1 mM of each primer, and 50 ng of DNA. One of the primers (forward or reverse) in each reaction was biotinilated to allow the purification in the next step (Additional file 1: Table S3 and Additional file 1: Table S4). Cycling conditions were: 45 cycles of 45 sec at 94°C, 45 sec at 56, 58 and 60°C for *GNRHR-SNP6*, *LHR-I499L*, and *IGF1-SnaBI *respectively, and 45 sec at 72°C, with a final elongation step of 7 min at 72°C. Biotinilated amplified products were purified by streptavidin-coated Sepharose beads capture to be used as pyrosequencing template. Pyrosequencing reaction was performed with 0.3 μM internal sequencing primer (Additional file 1: Table S4) and PyroMark Gold Q96 reagents (Qiagen, Hilden, Germany), and run on a PSQ96MA. Outgoing results were analyzed using pyrosequencing software (Biotage AB, Uppsala, Sweden).

#### Genetic variability measurements

Allele frequencies, unbiased expected heterozygosity (he) and Hardy-Weinberg equilibrium (HWE) for each SNP within each herd and for the whole sample were estimated using exact test implemented in GENEPOP 4 [29] and ARLEQUIN 3.5 [30] softwares. Phases were reconstructed with the Bayesian statistical method implemented in Phase v2.1.1 [31,32] using default options, except that the program was run five times and the last iteration was 10 times longer, as suggested by the authors. Phases were considered reliable when P value estimated by Phase was higher than 0.8.

#### Association between genetic markers and age at puberty

In male cattle, puberty is defined as the time when each ejaculation has at least 50 • 10^6 ^sperm cells and a 10% of linear motility [1]. In females, it is defined as the age at which the first *corpus luteum *is observed [33]. Routinely, in commercial farms, a bull is considered to arrive at puberty when its SC reaches to 28 cm. This trait has a high correlation with age at puberty [4]. For this reason, to perform the association analysis, SNPs and *GNRHR *haplotypes were tested against two estimated puberty ages. The first one considers the age when bulls reach 28 cm of SC, while the second one consider puberty age when M is 10% and C is 50 million. For the three measurements, the regression was performed with the following logistic equation using the NLIN procedure implemented into SAS 9.0 software (SAS Inst. Inc.) [34]:

$$\text{y}=\text{A}/\left[\text{1}+\text{ b }{\text{e}}^{\text{( -k t)}}\right]$$

y = measured traits (SC, M or C), t = age, A, b and k = parameters calculated for each individual from raw data. Furthermore, weight at 300 days was estimated with the following Gompertz equation using NLIN procedure implemented into SAS 9.0 software (SAS Inst. Inc.) [34]:

$$\text{y}=\text{A e}{\;}^{\text{[ - b e( -k t)]}}$$

The following general model was used to analyze the association between puberty traits and genotypes:

$${\text{Y}}_{\text{ijkl}}=\text{μ}+\;{\text{S}}_{\text{i}}\;+\;{\text{G}}_{\text{j}}+{\text{B}}_{\text{k}}+{\text{O}}_{\text{l}}+\text{β}\left({\text{x}}_{\text{ijkl}}-\text{x}\right)+\;{\text{e}}_{\text{ijk}},$$

where Y_ijkl _= phenotypic observation, μ = the overall mean, S_i _= the fixed effect of i^th ^year, G_j _= the fixed effect of j^th ^genotype, B_k _= the fixed effect of k^th ^herd, O_l _= random effect of l^th ^sire, β(x_ijkl_-x) = regression on body weight at 300 days and e_ijkl _= random error. The model used did not include a pedigree matrix because when the available pedigree data was considered, the fit estimation parameter (BIC) clearly increases. The statistical analyses were carried out utilizing MIXED procedure implemented into SAS 9.0 (SAS Inst. Inc.). Animals with missing data (field or genotype) were excluded from each analysis. For statistically significant (p < 0.05) and suggestive (p < 0.1) main effects, least squares means were reported and Bonferroni's means separation test was used to determine differences between genotypes. The percentage of the genetic variance accounted by the i-th SNP was computed according to Falconer and Markey [35].

## Competing interests

The authors declare that they have no competing interests.

## Authors' contributions

JPL, AJP, AB, GG conceptualized and supervised the whole study. AJP, AB and GG collected the phenotypic data. MEF, MVR, DEG and DMP performed DNA sequencing and SNP genotyping. JPL, ARM and GG analyzed the data. JPL, ARM, MEF, PPG and GG drafted the manuscript together. All authors read and approved the final manuscript.

## Supplementary Material

Additional file 1 **Table S1**. Gene frequency, Hardy-Weinberg equilibrium (HWE), expected heterozygosities (h_e_), and gene differentiation estimated for GNRHR-SNP5, GNRHR-SNP6, LHR-I499L and IGF1-SnaBI alleles identified by pyrosequencing analysis within each herd and in the whole sample. **Table S2**. Average estimated puberty age and weigh estimated at 300 days (W 300), with their standard errors (SE) calculated for each herd and year. Results for the two criterions for age of puberty are: i) estimated at Scrotal Circumference (SC) = 28 cm, ii) estimated at Sperm Motility (M) = 10% and Sperm Concentration (C) = 50 million. **Table S3**. Summary information of genotyped SNPs for gonadotropin-releasing hormone receptor (*GNRHR*). luteinizing hormone receptor (*LHR*) and insulin-like growth factor 1 (*IGF1*) genes. **Table S4**. Primer sequences and annealing temperatures used for SNP genotyping by pyrosequencing assays. Click here for file
