# Supplementary material for: Association between GNRHR, LHR and IGF1 polymorphisms and timing of puberty in male Angus cattle
Source: BMC Genet. 2012 Apr 5;13:26. doi: 10.1186/1471-2156-13-26 (PMC3344682; doi:10.1186/1471-2156-13-26)
Supplement: Additional file 1 — Table S1. Gene frequency, Hardy-Weinberg equilibrium (HWE), expected heterozygosities (he), and gene differentiation estimated for GNRHR-SNP5, GNRHR-SNP6, LHR-I499L and IGF1-SnaBI alleles identified by pyrosequencing analysis within each herd and in the whole sample. Table S2. Average estimated puberty age and weigh estimated at 300 days (W 300), with their standard errors (SE) calculated for each herd and year. Results for the two criterions for age of puberty are: i) estimated at Scrotal Circumference (SC) = 28 cm, ii) estimated at Sperm Motility (M) = 10% and Sperm Concentration (C) = 50 million. Table S3. Summary information of genotyped SNPs for gonadotropin-releasing hormone receptor (GNRHR). luteinizing hormone receptor (LHR) and insulin-like growth factor 1 (IGF1) genes. Table S4. Primer sequences and annealing temperatures used for SNP genotyping by pyrosequencing assays. [file 1471-2156-13-26-S1.DOC]

**Supplementary Material**

**Table S1.** Gene frequency, Hardy–Weinberg equilibrium (HWE), expected heterozygosities (h_e_), and gene differentiation estimated for GNRHR-SNP5, GNRHR-SNP6, LHR-I499L and IGF1-SnaBI alleles identified by pyrosequencing analysis within each herd and in the whole sample.

| Polymorphism | Population | Gene frequencies | | | | | F_IS_ - p | h_e_ |
| --- | --- | --- | --- | --- | --- | --- | --- | --- |
| GNRHR-SNP5 |  | A | | | G | |  |  |
|  | Herd 1 | 0.42 | | | 0.58 | | 0.313 | 0.488 |
|  | Herd 2 | 0.57 | | | 0.43 | | 0.804 | 0.494 |
|  | Combined | 0.46 | | | 0.54 | | 0.620 | 0.497 |
| GNRHR-SNP6 |  | C | | | T | |  |  |
|  | Herd 1 | 0.38 | | | 0.62 | | 0.302 | 0.471 |
|  | Herd 2 | 0.28 | | | 0.72 | | 0.070 | 0.407 |
|  | Combined | 0.35 | | | 0.65 | | 0.794 | 0.458 |
| LHR-I499L |  | A | | | C | |  |  |
|  | Herd 1 | 0.28 | | | 0.72 | | 0.865 | 0.407 |
|  | Herd 2 | 0.10 | | | 0.90 | | 1.000 | 0.179 |
|  | Combined | 0.24 | | | 0.76 | | 0.618 | 0.366 |
| IGF1-SnaBI |  | C | | | T | |  |  |
|  | Herd 1 | 0.55 | | | 0.45 | | 0.261 | 0.497 |
|  | Herd 2 | 0.60 | | | 0.40 | | 1.000 | 0.484 |
|  | Combined | 0.56 | | | 0.44 | | 0.388 | 0.494 |
|  |  | GC | GT | AC | | AT |  |  |
| GNRHR_hap | Herd 1 | 0.34 | 0.24 | 0.04 | | 0.38 | **0.002** | 0.679 |
|  | Herd 2 | 0.23 | 0.20 | 0.05 | | 0.52 | 0.080 | 0.643 |
|  | Combined | 0.31 | 0.23 | 0.04 | | 0.42 | **0.006** | 0.675 |

|  |
| --- |

Significant F_IS_ – p values (P > 0.05) were remarked in bold.

**Table S2.** Average estimated puberty age and weigh estimated at 300 days (W 300), with their standard errors (SE) calculated for each herd and year. Results for the two criterions for age of puberty are: i) estimated at Scrotal Circumference (SC) = 28 cm, ii) estimated at Sperm Motility (M) = 10% and Sperm Concentration (C) = 50 million

| **Herd - Year** | **N = 277** | **Age at SC28** | **Age at 5 10^7^ / 10% M** | **W 300** |
| --- | --- | --- | --- | --- |
| 1 – 2009 | 94 | 265.03 ± 21.54 | 283.22 ± 33.44 | 259.72 ± 20.45 |
| 1 – 2010 | 112 | 276.28 ± 23.59 | 269.37 ± 37.22 | 285.61 ± 25.44 |
| 2 – 2009 | 71 | 308.44 ± 35.47 | 296.29 ± 40.53 | 241.26 ± 29.12 |

**Table S3.** Summary information of genotyped SNPs for gonadotropin-releasing hormone receptor (*GNRHR*). luteinizing hormone receptor (*LHR*) and insulin-like growth factor 1 (*IGF1*) genes.

| SNP | Gene | Chromosome | Nucleotide position - Polymorphisms – amino acid site | Gene region | Effect |
| --- | --- | --- | --- | --- | --- |
| GHRHR-SNP5 | GNRHR | BTA6 | NW_001495209.1^1^:g. 884033T>C – aa137 | Exon 1 | Silent |
| GNRHR-SNP6 | GNRHR | BTA6 | NW_001495209.1^1^:g. 871174C>T – aa236 | Exon 2 | Silent |
| LHR-I499L | LHR | BTA11 | NM_174381.1^2^ c. 1504 A>C – aa499 | Exon 11 | Non-synonimous (I>L) |
| IGF1-SnaBI | IGF1 | BTA5 | AF017143.1^3^:g. 512T>C | Promoter | - |

^1^ *Bos taurus* chromosome 6 genomic contig (NW_001495209.1/ Bt6_WGA699_4), reference assembly (based on Btau_4.0, <http://www.hgsc.bcm.tmc.edu/projects/bovine/>).

^2^ NM_174381.1 Bos taurus luteinizing hormone/choriogonadotropin receptor (*LHCGR*), mRNA.

^3^ AF017143.1 Bos taurus insulin-like growth factor-I gene, 5' flanking region and partial cds.

**Table S4.** Primer sequences and annealing temperatures used for SNP genotyping by pyrosequencing assays.

| SNP | Primer | Primer sequence | Annealing temperature | Assay reference |
| --- | --- | --- | --- | --- |
| GHRHR-SNP5^1^ | GNRHr-E1-F (Biotilinated) | 5´-CACTGGATGGAATGTGGAACA-3´ | 63 | Lirón et al. 2010 |
|  | GnRHr-E1-R | 5´-CTGTGGTCCAGCAAAGATGC-3´ |  |  |
|  | GnRHr-SNP5-sec | 5'-CGCCAGCGAGCGGTC-'3 |  |  |
| GNRHR-SNP6^1^ | GnRHr-SNP6-F | 5´-CAGCTGCCTCTTCATCATCC-3´ | 56 | Present work |
|  | GnRHr-SNP6-R (Biotilinated) | 5´-TGCCTCATAGGGTGATTTTGA-3´ |  |  |
|  | GnRHr-SNP6-sec | 5´-ATGCAAAAATCATCTT-3´ |  |  |
| LHR-I499L^2^ | LHR-F (Biotilinated) | 5´- AGACTGGCAGACAGGGAGTG -3´ | 58 | Present work |
|  | LHR-R-pyro-int | 5'- AGTGAAAAAGCCAGCC-3' |  |  |
|  | LHr-piroint-Angus | 5´-GCATAGGTGATGGTGTG-3´ |  |  |
| IGF1-SnaBI^3^ | IGF1-prom-F | 5´-CCAGCGCTGTCTTCCATTCTA-3´ | 60 | Present work |
|  | IGF1-prom-R (Biotinilated) | 5´-TGATTAACTTTCTACCGGGCG-3´ |  |  |
|  | IGF1-prom-seq | 5´-ATTGCTCGCCCATCCTC-3´ |  |  |

^1^ SNPs reported in Lirón *et al* (2010); ^2^ SNPs reported in the present work; ^3^ SNPs reported in Ge *et al* (2001).
